# Metabolic syndrome and cognitive performance across the adult lifespan

**DOI:** 10.1371/journal.pone.0249348

**Published:** 2021-05-06

**Authors:** Lori Haase Alasantro, Tracey H. Hicks, Erin Green-Krogmann, Claire Murphy

**Affiliations:** 1 San Diego Joint Doctoral Program in Clinical Psychology, San Diego State University/University of California, San Diego, California, United States of America; 2 Department of Psychiatry, University of California, San Diego, La Jolla, California, United States of America; 3 Department of Psychology, San Diego State University, San Diego, California, United States of America; Nathan S Kline Institute, UNITED STATES

## Abstract

Metabolic Syndrome (MetS) is associated with increased rates of mortality and increased risk for developing dementia. Changes in brain structure and cognitive functioning have been reported within the literature. However, research examining cognitive performance in individuals with MetS is limited, inconclusive, and focuses primarily on older cohorts. As such, the effect of MetS on cognitive functioning earlier in the lifespan is unclear. This study aimed to investigate cognitive performance in young, middle-aged, and older adults with multiple metabolic and vascular risk factors in a sample of community dwelling participants (N = 128). Participants were administered a comprehensive neuropsychological battery and self-report measures. As expected, older adults performed more poorly than young and middle-aged adults across most assessments. Relative to controls, individuals with MetS reported greater hunger and disinhibited eating. MetS participants performed more poorly on Color-Word Interference: Inhibition. Additionally, when weight was accounted for, there was a significant relationship between MetS and select executive functioning tasks in middle-aged adults. These findings suggest that aspects of executive functioning may be impaired in MetS and could be further impacted by excess weight in middle-age. Future studies aimed at investigating potential causal relationships between metabolic and vascular risk factors, disinhibited eating, and executive dysfunction may provide insight into effective intervention targets to prevent MetS.

## Introduction

Metabolic syndrome (MetS) is a constellation of vascular and metabolic risk factors that are directly related to the development of Cardiovascular Disease (CVD) and increase the risk of developing type 2 diabetes mellitus (DM) [1–3]. These vascular and metabolic risk factors frequently occur in combination, and taken together, increase CVD morbidity rates more than the individual components alone [4, 5]. Middle-aged and older adults with MetS are three to four times more likely to have coronary heart disease, stroke, and higher mortality [4–7].

The prevalence rate of MetS within the United States was estimated to be 33% of the population between 2003 and 2012 [8]. Older adults (>65 years of age) are at an increased risk for CVD, type 2 DM, and MetS [9, 10]; however, these conditions have also been documented in young adulthood [11, 12]. While the long-term deleterious effects of developing these conditions in young adulthood are not well-established, the presence of multiple vascular risk factors and MetS in middle-aged and older adults, increases the risk of CVD and mortality [5, 10], the risk of developing dementia [13–16], and has been reported to be associated with impairments in executive functioning [17–19].

Obesity—a crucial component of MetS—is also highly prevalent, with approximately a third of the world’s population classified as overweight or obese [20]. Obesity is associated with increased risk for the development of MetS over the lifespan due its significant cardiovascular impact [21]. Furthermore, there is evidence suggesting that obesity is associated with cognitive deficits across the lifespan [22–25]. More specifically, significant deficits in decision making, cognitive flexibility, and inhibition have been linked to excess weight or obesity [22, 23, 26–28]. One study found deficits in inhibitory control and cognitive flexibility significantly predicted body weight in primary school children, though the direction of the effect warrants further study [29]. Interestingly, a review by Smith and colleagues [30], noted two studies that suggested that in older adults, obesity predicted better cognitive abilities or less decline in function; these findings may indicate a protective role of weight or a survival effect in older ages (i.e., middle-aged obesity-related mortality). Thus, components of executive functioning may represent the earliest domains of cognitive change associated with metabolic and vascular risk factors.

MetS has been associated with poorer cognitive performance [31–34]. Focal deficits in executive functioning (specifically cognitive flexibility and inhibition) have been repeatedly demonstrated in MetS [17–19]. However, specific neuropsychological domains of impairment have largely been inconsistent across the literature [35, 36]. Although cross-sectional studies examining domain-specific aspects of cognitive function have revealed poorer performance in MetS relative to controls on measures of information processing speed [19, 37, 38], attention [38], verbal memory [32, 37], executive functioning [19, 38], and fluid intelligence [37], there is considerable variability in the pattern of cognitive decline [39] and type of assessment used to measure each domain [35, 36].

To date, the majority of these studies have focused primarily on middle-aged and older adults; as such, information regarding the effect of MetS on cognition across the lifespan is limited. For instance, despite the increased risk of multiple cardiovascular risk factors in young adulthood [11], the effect of MetS on cognitive function in young adults has hardly been examined. Regarding middle-aged adults with MetS, those who consistently met criteria over a 10-year period, performed significantly poorer than those with non-persistent MetS and those without any history of MetS on measures of memory, verbal fluency, reasoning, and vocabulary [40]. In the oldest age group (85+ years of age), MetS has not been associated with significant declines in cognitive performance [35, 41], which might suggest that some aspects of MetS may be protective against cognitive decline later in life.

In the current study, we used the Color-Word Inhibition, Trails, Verbal Fluency and Design Fluency subtests of the Delis-Kaplan Executive Function System (D-KEFS) [42]; the California Verbal Learning Test-II (CVLT-II) [43]; Brief Visuospatial Memory Test-Revised (BVMT-R) [44]; and Conners’ Continuous Performance Test-2 (CPT-2) [45] to examine cognitive differences in young, middle-aged and older adults with and without vascular and metabolic risk factors. Investigating the effects of MetS on cognition in young, middle-aged, and older adults would help elucidate the age group in which changes in cognition first appear in MetS and may provide support for initiating targeted interventions earlier in the lifespan.

## Method

### Participants

Participants were part of a larger research study aimed at investigating the relationship among chemosensory and cognitive processes in healthy aging and metabolic disease. Participants received monetary compensation. This study included young adults (18–35 years of age, n = 42), middle-aged adults (45–54 years of age, n = 41), and older adults (65–86 years, n = 45), totaling at 128. Participants were excluded if they were left-handed, had a positive history of head injury with loss of consciousness > 5 minutes, substance use disorders, neurological or psychiatric diseases, or if they scored less than 24 on the MMSE, or less than 130 on the DRS. The research was approved by the San Diego State University Human Research Protections Program (1633) and the University of California, San Diego Human Research Protections Program (170289). Subjects provided written consent.

The following inclusion criteria were used to determine metabolic status. According to the International Diabetes Federation [3] and subsequent modification [46], the diagnosis of MetS requires ≥ 3 of 5 of the following risk factors: central obesity, operationally defined as body mass index (BMI) >30kg/m^2^ or waist circumference ≥ 94 cm for males and 80 cm for females; raised triglycerides (≥ 150 mg/dL) or currently receiving treatment for dyslipidemia; reduced HDL cholesterol (< 40 mg/dL in males and < 50 mg/dL in females) or currently receiving treatment for dyslipidemia; raised blood pressure (BP; systolic BP ≥ 130 or diastolic BP ≥ 85 mm Hg) or treatment of diagnosed hypertension; and raised fasting plasma glucose (≥ 100 mg/dL) or previous diagnosis of type 2 diabetes. Ethnic specific values of waist circumference were employed as outlined by the IDF (IDF, 2006). Blood pressure, height, weight, waist circumference, and systolic/diastolic blood pressures were measured. Calculations were performed for pulse pressure (systolic—diastolic blood pressure) and BMI (kg/m^2^). Participants’ self-reported a diagnosis and/or current treatment for raised triglycerides, reduced HDL, and type 2 DM.

Based on the MetS criteria outlined above, individuals were classified as either having MetS or as normal controls (Table 1). For the young adult metabolic cohort, all participants met criteria for obesity. Prevalence of MetS in young adults is estimated to be 20.3% and 15.6% for male and females, respectively [10]. For the present young adult metabolic cohort, 59% of participants met full criteria (3 out of 5 risk factors), 18% met partial criteria (2 out of 5 risk factors), and 23% were classified as only obese. Obesity is associated with increased risk for the development of MetS over the lifespan [47]. As such, for the purpose of the present manuscript, the metabolic young cohort will be operationally defined as obese with additional risk factors.

**Table 1:** Demographic characteristics of participants.

	Age Group & Metabolic Status: Mean (Standard Error)					
Variable	Young Control (n = 20)	Young Metabolic (n = 22)	Middle-age Control (n = 18)	Middle-age Metabolic (n = 23)	Older Control (n = 22)	Older Metabolic (n = 23)
Age	23.80 (.90)	25.27 (.90)	49.33 (.76)	50.35 (.65)	72.23 (1.75)	69.57 (1.45)
Education	15.35 (.35)	15.32 (.54)	15.11 (.59)	14.74 (.48)	14.73 (.54)	14.52 (.55)
Gender (% Male)	35	36.4	44.4	30.4	59.1	39.1
MMSE	29.63 (.22)	29.44 (.17)	29.39 (.20)	28.83 (.35)	28.52 (.36)	28.43 (.27)
DRS	141.22 (.46)	141.71 (1.08)	141.39 (.56)	140.74 (.48)	140.41 (.86)	141.39 (.63)
**Body Measurements**						
Weight (lbs)	147.98 (6.71)	223.56 (7.75)	163.47 (3.25)	254.14 (6.79)	156.25 (6.13)	201.87 (8.71)
Height (cm)	171.83 (2.56)	170.46 (1.47)	171.59 (2.14)	168.68 (1.89)	170.00 (2.88)	166.03 (1.90)
BMI	22.52 (.57)	34.74 (1.08)	25.04 (.61)	40.27 (1.21)	24.61 (.61)	33.10 (1.35)
Waist Circumference (cm)	79.79 (2.23)	106.88 (2.66)	91.57 (2.92)	121.89 (2.11)	89.87 (2.01)	109.35 (2.79)
Systolic Blood Pressure	119.52 (3.07)	124.24 (2.69)	124.93 (4.61)	138.44 (2.66)	145.45 (5.10)	140.28 (3.86)
Diastolic Blood Pressure	70.78 (2.33)	75.01 (1.65)	76.85 (2.83)	83.85 (1.67)	75.11 (2.50)	72.33 (1.92)
Pulse Pressure	48.74 (2.45)	50.75 (1.80)	48.08 (3.18)	54.58 (1.92)	70.34 (3.98)	67.93 (4.07)
Stroke Risk (%)	2.40 (.34)	2.67 (.25)	2.89 (.33)	4.70 (.66)	12.05 (1.66)	14.87 (1.97)

*Note*. MMSE = Mini-mental Status Examination; DRS = Dementia Rating Scale.

BMI = body mass index; lbs = pounds, cm = centimeters.

### Procedures

Participants underwent two separate testing sessions, each session lasting approximately 2 hours. In the first session, participants were administered measures of general cognitive functioning and questionnaires regarding their metabolic status. During the second session, neuropsychological measures were administered.

### Cognitive measures

The following tests were administered as part of a larger test battery.

#### Mini-Mental State Exam (MMSE)

The MMSE is a brief measure of cognition [48]. It is commonly administered to older adults as a screen for cognitive impairment and to track changes in cognition over time.

#### Dementia Rating Scale (DRS)

The DRS is a global measure of cognition that can be administered to older adults with known or suspected dementia [49]. The total scores from the MMSE and DRS were used to exclude those whose scores enter the clinically impaired range, defined as less than 24 for the MMSE and less than 130 for the DRS.

#### Subtests from the Delis-Kaplan Executive Function System (D-KEFS)

The D-KEFS is a comprehensive set of tests aimed at assessing higher-level cognitive functions [42]. The Verbal Fluency subtest measures verbal response generation and cognitive flexibility [42]. The Design Fluency subtest measures non-verbal response generation, inhibition, and cognitive flexibility [42]. The Color-Word Interference subtest assesses cognitive flexibility and inhibition [42]. It is based on the Stroop Color and Word Task [50] and consists of four conditions: Color Naming, Word Reading, Inhibition, and Inhibition Switching. Trails is designed to assess cognitive flexibility and executive functioning on a visual-motor task [42].

#### Conners’ Continuous Performance Test (CPT-2)

The CPT-2 assesses sustained attention, reaction time, and impulsivity [45].

#### California Verbal Learning Test- second edition (CVLT-II)

The CVLT-II is designed to measure verbal learning, short- and long-term memory, cued recall, and recognition [43].

#### Brief Visuospatial Memory Test-Revised (BVMT-R)

The BVMT-R assesses visuospatial learning, memory, and recognition [44].

### Self-report questionnaires

Self-report questionnaires were administered to assess mood and impulsive personality traits. Specifically, the Beck Depression Inventory—Second Edition (BDI) [51] was used to assess depressive symptoms; the State Trait Anxiety Inventory (STAI) [52] was used to screen for anxiety symptoms/traits; The Three-Factor Eating Questionnaire (TFEQ) [53] was used to assess food intake-behavior, including disinhibition; and the Barratt Impulsiveness Scale (BIS) [54] was used to assess impulsiveness. The percentage of stroke risk was assessed using the Stroke Risk Assessment Test [55].

### Statistical analyses

#### Self-report measures

Multivariate analyses of covariance (MANCOVAs) were performed to examine the potential associations of age group and metabolic status, with self-report measures, while controlling for gender and years of education, conducted in the following groupings: 1) BDI and STAI (state and trait indices); 2) TFEQ (cognitive restraint, disinhibition, hunger), and 3) BIS (first order factors: attention, motor, self-control, cognitive complexity, perseverance, cognitive instability).

#### Cognitive measures

Individual measures were analyzed using raw scores. MANCOVAs were conducted to examine potential associations of age group and metabolic status with cognitive functioning while accounting for gender and education level. The following indices were investigated in the following MANCOVA groupings: 1) DRS total score, MMSE total score, Digit span total score (due to significant correlations between these variables p < .01); 2) BVMT-R: total and delayed scores; 3) CVLT-II: total score of trials 1–5, short delay free and cued recall, and long delay free and cued recall; 4) CPT-2: clinical percentage, non-clinical percentage, omission, commission, variability, response style, and perseveratives; 5) D-KEFS Verbal Fluency: letter, category, category switching, switching accuracy, and set-loss errors; 6) D-KEFS Design Fluency: filled dots, empty dots, and switching;7) D-KEFS Trails: visual scanning, number sequencing, letter sequencing, number-letter switching, and motor speed; and 8) D-KEFS Color-Word Interference: color naming, word reading, inhibition, switching, and switching errors.

An alpha level of p = .05 was used for all analyses to achieve a balance between small sample size and Type I and Type II errors. Bonferroni post-hoc tests were used to probe the significant effects at an alpha of.05.

#### Exploratory analyses

As a follow-up to the main analysis, the raw data on cognitive measures were re-analyzed in MANCOVAs conducted to examine the role of weight in the context of metabolic status. As the middle-age group displayed higher mean weight than the other two age groups, we conducted MANCOVAs separated by age group to evaluate the relationship between metabolic status while controlling for weight, gender, and education level.

## Results

### Self-report measurements

MANCOVAs did not demonstrate significant interactions between age group and metabolic status on self-report measures while controlling for gender and education levels (Table 2); however, there were significant differences by age group and metabolic status for several measures. There was a main effect of age group on the BIS: Self-control ([*F*(2,113) = 4.05, p = .02]; Table 2); Bonferroni analyses revealed that middle-aged adults had significantly higher scores on this measure as compared to older adults (S1 Table). There was a main effect of metabolic status in which individuals with MetS had significantly higher scores on the BDI, TFEQ: disinhibition, and TFEQ: hunger as compared to controls (BDI [*F*(1,107) = 4.81, p = .04], TFEQ: disinhibition [*F*(1,118) = 29.46, p < .001], and TFEQ: hunger [*F*(1,118) = 14.87, p < .001], Table 2).

**Table 2:** Self-report measurements of participants.

	Age Group & Metabolic Status: Mean (Standard Error)					
Variable	Young Control	Young Metabolic	Middle-age Control	Middle-age Metabolic	Older Control	Older Metabolic
BDI-II	5.15 (.88)	8.75* (2.21)	7.06 (2.62)	8.00* (1.72)	4.42 (.85)	9.30* (1.87)
STAI: State	29.75 (1.58)	31.17 (2.20)	31.89 (2.92)	31.00 (1.67)	27.58 (1.76)	32.83 (2.39)
STAI: Trait	34.55 (1.67)	37.00 (2.78)	33.89 (2.72)	32.35 (1.79)	30.42 (1.70)	35.65 (2.52)
TFEQ: Cognitive Restraint	9.40 (1.00)	9.59 (1.09)	10.00 (1.18)	8.04 (.90)	10.62 (1.34)	10.17 (.83)
TFEQ: Disinhibition	5.05 (.69)	7.41*** (.72)	4.17 (1.05)	9.52*** (.78)	4.24 (.72)	7.04*** (.72)
TFEQ: Hunger	3.95 (.65)	4.73*** (.62)	3.61 (.69)	7.09*** (.70)	3.29 (.57)	5.52*** (.67)
BIS: Attention	9.90 (.57)	9.85 (.51)	9.39 (.37)	9.55 (.51)	9.57 (.54)	10.35 (.60)
BIS: Motor	14.10 (.58)	13.65 (.79)	14.11 (.84)	15.40 (.67)	13.81 (.46)	13.43 (.54)
BIS: Self-control	12.05 (.85)	11.15 (.69)	13.06* (.72)	13.3* (.82)	10.67 (.76)	11.39 (.74)
BIS: Cognitive Complexity	11.70 (.55)	10.15 (.47)	11.56 (.66)	11.80 (.49)	12.00 (.43)	11.26 (.61)
BIS: Perseverative	7.25 (.37)	7.00 (.41)	7.89 (.46)	8.05 (.49)	7.19 (.29)	7.61 (.41)
BIS: Cognitive Instability	5.70 (.411)	6.40 (.43)	5.67 (.26)	5.10 (.37)	5.24 (.34)	5.96 (.43)
BIS: First order factors (total)	60.65 (2.24)	58.20 (2.21)	61.67 (2.27)	63.20 (2.55)	58.48 (1.66)	60.00 (2.09)

Note. BDI = Beck Depression Inventory-II; STAI = State Trait Anxiety Inventory; TFEQ = Three-Factor Eating Questionnaire; BIS = Barratt Impulsiveness Scale.

### Cognitive measures

#### Relationship between age group and cognitive functioning

MANCOVAs demonstrated significant main effects of age group on the MMSE, D-KEFS Verbal Fluency: category, switching, and switching accuracy; D-KEFS Design Fluency: filled dot, empty dot, and switching conditions; D-KEFS Trails: visual scanning, number sequencing, letter sequencing, number-letter switching, and motor speed; D-KEFS Color-word interference: color naming, word reading, inhibition, and inhibition switching; CVLT-II: total recall, short delay free and cued recall, and long delay free and cued recall; BVMT-R: total recall and long delay recall; digit span total; and CPT-2 clinical percentage and non-clinical percentage (Tables 3 and 4).

**Table 3:** Raw score means and standard error of cognitive performance for the main effect of age group.

	Age Group					
	Young	Middle-age			Older	
Raw Scores	Mean (Standard Error)					F
**MMSE**						
	29.54 (.14)		29.07 (.22)	28.48 (.22)		5.05**
**Verbal Fluency**						
Letter	42.95 (1.89)	41.91 (2.18)			42.46 (1.97)	.07
Category	45.56 (1.19)	43.60 (1.27)			40.46 (1.33)	4.87**
Switching	15.18 (.45)	15.51 (.42)			13.14 (.47)	8.99***
Switching Accuracy	14.28 (.50)	14.69 (.51)			12.14 (.53)	8.12***
Set loss	.54 (.15)	1.14 (.27)			1.79 (.32)	5.75**
**Design Fluency**						
Filled Dot	11.15 (.56)	10.94 (.65)			8.98 (.61)	3.69*
Empty Dot	12.00 (.50)	12.51 (.67)			10.14 (.62)	5.09**
Switching	9.56 (.39)	8.54 (.42)			6.98 (.42)	10.14***
Set Loss	1.90 (.44)	2.20 (.44)			3.19 (.50)	1.45
**Trails**						
Visual Scanning	18.77 (.91)	19.31 (.70)			24.67 (1.13)	12.52***
Number Sequencing	25.36 (1.19)	28.71 (1.37)			41.26 (3.24)	14.09***
Letter Sequencing	25.79 (1.36)	30.34 (1.61)			41.72 (2.67)	16.72***
Number-Letter Switching	58.46 (2.87)	75.17 (5.06)			110.19 (8.32)	20.36***
Motor Speed	22.15 (.98)	25.69 (1.36)			33.93 (2.46)	11.89***
**Color-Word Interference**						
Color Naming	28.46 (.89)	30.80 (1.05)			32.26 (1.14)	3.79*
Word Reading	21.21 (.73)	22.74 (.79)			24.43 (.97)	4.22*
Inhibition	47.56 (2.44)	54.09 (2.12)			68.02 (2.97)	18.70***
Inhibition Switching	53.13 (1.47)	60.11 (3.22)			71.21 (4.26)	8.20***

*Note*.

** = p < .01;

* = p < .05.

**Table 4:** Raw score means and standard error of cognitive performance for the main effect of age group.

	Age Group			
	Young	Middle-age	Older	
Raw Scores	Mean (Standard Error)			F
**CVLT-II**				
Total List Learning	54.24 (1.31)	53.71 (1.45)	46.79 (1.90)	7.74***
Short Delay Free Recall	11.87 (.42)	11.80 (.51)	9.33 (.56)	9.30***
Short Delay Cued Recall	12.50 (.46)	12.94 (.42)	10.83 (.48)	6.69**
Long Delay Free Recall	12.39 (.46)	12.66 (.50)	9.71 (.55)	11.50***
Long Delay Cued Recall	12.76 (.79)	13.06 (.45)	10.93 (.53)	6.84**
**BVMT-R**				
Total Learning	27.37 (.79)	23.42 (1.16)	20.46 (1.18)	11.02***
Delayed Recall	10.32 (.25)	9.39 (.44)	8.51 (.47)	5.26**
**Digit Span**				
Total	20.23 (.69)	18.20 (.64)	16.60 (.58)	8.17***
**CPT-2**				
Omission	2.08 (.58)	13.35 (5.01)	7.54 (2.02)	1.00
Commission	13.21 (1.40)	17.86 (2.97)	11.88 (1.04)	.38
Response Time	378.66 (10.40)	338.06 (25.86)	436.29 (12.40)	.56
Variability	8.15 (.75)	16.92 (3.48)	9.67 (1.10)	.12
Perseveratives	.72 (.25)	15.61 (7.22)	1.20 (.52)	.10
Clinical %	41.21 (3.66)	53.50 (3.03)	63.14 (3.41)	8.54***
Non-Clinical %	58.79 (3.66)	46.33 (3.06)	36.86 (3.41)	8.51***
Response Style	.60 (.11)	10.68 (3.48)	1.35 (.278)	3.44*

*Note*.

** = p < .01;

* = p < .05.

CVLT-II = California Verbal Learning Test-II, BVMT-R = Brief Visual Memory Test-Revised, and CPT-2 = Conner’s Continuous Performance Test-II.

#### Relationship between metabolic status and cognitive functioning

**Fig 1:**
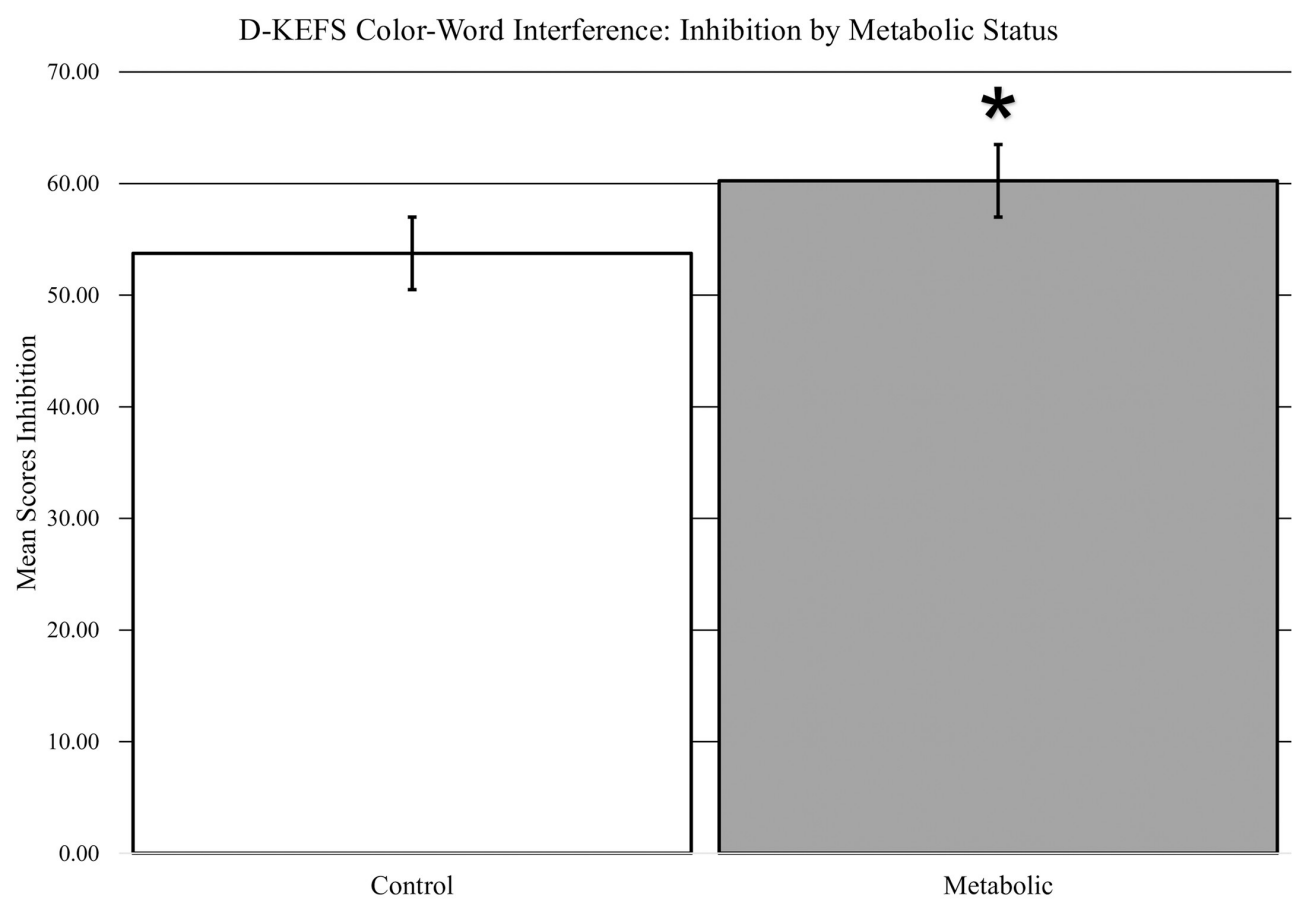
Main effect of metabolic syndrome on cognitive inhibition. * indicates p < .05.

The MANCOVAs displayed a significant main effect of metabolic status on a single measure of executive functioning, Color-Word Interference Inhibition, in which participants with MetS took more time to complete the task as compared to controls (Fig 1, [*F*(1,107) = 6.14, p = .015]). There were no other significant main effects of metabolic status (Table 5).

**Table 5:** Status effects.

	Control	MetS	
	Mean (Standard Error)		F
**TFEQ**			
Cognitive Restraint	10.02 (.68)	9.26 (.55)	.53
Disinhibition	4.49 (.47)	8.00 (.44)	26.46***
Hunger	3.61 (.36)	5.79 (.40)	14.87***
**BIS**			
Attention	9.63 (.29)	9.94 (.31)	.55
Motor	14.00 (.36)	14.13 (.39)	2.04
Self-Control	11.86 (.46)	11.92 (.44)	.00
Cognitive Complexity	11.76 (.31)	11.08 (.32)	2.93
Perseverative	7.42 (.21)	7.56 (.25)	.11
Cognitive Instability	5.53 (.20)	5.83 (.24)	.79
**MMSE**			
Total	29.16 (.17)	28.86 (.17)	.93
**DRS**			
Total	140.97 (.39)	141.22 (.39)	1.07
**Digit Span**			
Total	18.47 (.62)	18.10 (.48)	.05
**CPT-2**			
Omission	14.89 (4.25)	17.60 (4.16)	.11
Commission	19.38 (2.81)	19.28 (2.41)	.03
Response Time	328.42 (23.13)	330.94 (22.56)	.01
Variability	17.87 (2.91)	18.43 (3.04)	.03
Perseveratives	12.27 (3.36)	15.07 (5.32)	.22
Clinical %	51.78 (3.39)	53.56 (2.87)	.07
Non-Clinical %	48.10 (3.40)	46.44 (2.87)	.08
Response Style	12.37 (3.29)	12.76 (3.37)	.00
**CVLT-II**			
Total Trials 1–5	51.47 (1.47)	51.23 (1.29)	.04
SDFR	11.07 (.44)	10.77 (.44)	.37
SDCR	11.81 (.42)	12.25 (.35)	.62
LDFR	11.47 (.46)	11.52 (.44)	.00
LDCR	11.90 (.44)	12.48 (.36)	1.11
**BVMT-R**			
Total Trials 1–3	24.00 (.83)	23.35 (1.05)	.27
Delay	9.70 (.28)	9.05 (.39)	1.89
**D-KEFS Verbal Fluency**			
Letter	42.81 (1.60)	42.10 (1.66)	.09
Category	42.39 (1.03)	43.83 (1.11)	.74
Switching	14.78 (.38)	14.28 (.41)	1.25
Switching accuracy	13.92 (.40)	13.31 (.48)	1.44
Set-loss errors	1.03 (.21)	1.33 (.24)	.91
**D-KEFS Design Fluency**			
Filled Dots	10.85 (.52)	9.72 (.49)	2.33
Empty Dots	12.02 (.49)	10.91 (.52)	3.31
Switching	8.31 (.34)	8.31 (.39)	.00
Set-loss errors	2.39 (.36)	2.53 (.42)	.01
**D-KEFS Trails**			
Visual Scanning	21.41 (.85)	20.79 (.87)	.17
Number Sequencing	32.51 (2.21)	31.90 (1.93)	.01
Letter Sequencing	32.85 (2.02)	33.17 (1.77)	.06
Switching	82.25 (6.45)	82.69 (5.00)	.05
Motor Speed	27.22 (1.83)	27.86 (1.35)	.19
**Color-Word Interference**			
Color Naming	29.75 (.83)	31.37 (.91)	2.22
Word Reading	22.15 (.69)	23.54 (.72)	2.55
Inhibition	53.75 (2.16)	60.25 (2.56)	6.14*
Inhibition Switching	61.53 (2.65)	62.05 (3.04)	.08
Inhibition Switching errors	1.64 (.31)	1.60 (.26)	.09

*Note*.

*** = p < .001;

** = p < .01;

* = p < .05.

#### Relationship among age group, metabolic status, and cognitive functioning

There were significant interactions between age group, metabolic status, and cognitive functioning on the CPT-2: commission, response time, variability, perseveratives, and response styles sub-measures (Table 6). However, there were no additional significant interactions between age group and metabolic status when controlling for gender and education level (refer to Tables 7–9 for mean cognitive scores).

**Table 6:** Metabolic status by age group interactions for CPT-2.

CPT-2	F
Omission	2.56
Commission	4.20*
Response Time	3.64*
Variability	4.28*
Perseveratives	3.58*
Clinical %	1.19
Non-Clinical %	1.23
Response Style	3.44*

*Note*.

** = p < .01;

* = p < .05.

**Table 7:** Relationship between age, metabolic status, and cognitive functioning.

	Age Group & Metabolic Status: Mean (Standard Error)					
Variable	Young Control	Young Metabolic	Middle-age Control	Middle-age Metabolic	Older Control	Older Metabolic
**Digit Span**						
Total	21.45** (1.10)	18.95 (.74)	17.82 (.92)	18.56 (.90)	16.27 (.83)	16.95 (.81)
**CPT-2**						
Omission	1.35 (.48)	2.84 (1.07)	7.46 (3.98)	11.08 (4.57)	6.00 (2.26)	9.00 (3.34)
Commission	17.33 (4.31)	26.81 (5.42)	14.69 (3.42)	19.00 (4.64)	28.20 (6.88)	15.24 (2.72)
Response Time	385.21 (15.57)	371.77* (13.94)	361.94 (31.04)	330.08 (40.68)	452.36 (18.97)	420.98 (15.82)
Variability	7.07 (.94)	9.28 (1.13)	13.95 (3.36)	15.63 (4.66)	10.80 (2.04)	8.58 (.91)
Perseveratives	.60 (.28)	.84 (.44)	5.93 (3.66)	12.31 (5.45)	2.05 (1.02)	.38* (.20)
Clinical Percentage	37.10 (5.04)	45.54 (5.28)	56.49 (4.64)	47.78 (2.88)	68.31 (4.95)	58.21 (4.55)
Non-Clinical Percentage	62.90 (5.04)	54.46 (5.28)	43.16 (4.68)	52.22 (2.88)	31.69 (4.95)	41.79 (4.55)
Response Style	.61 (.13)	.60 (.18)	7.05 (4.40)	11.75 (5.07)	1.44 (.42)	1.26 (.38)

***Note***. CPT-2 = Conner’s Performance Test-II. Significant exploratory analyses are denoted here using *** = p < .001;

** = p < .01;

* = p < .05.

**Table 8:** Relationship between age, metabolic status, and cognitive functioning.

	Age Group & Metabolic Status: Mean (Standard Error)					
Variable	Young Control (n = 20)	Young Metabolic (n = 22)	Middle-age Control (n = 18)	Middle-age Metabolic (n = 23)	Older Control (n = 22)	Older Metabolic (n = 23)
**Color-Word Interference**						
Color Naming	27.90 (1.05)	29.05 (1.49)	29.76 (1.56)	31.78 (1.41)	31.41 (1.56)	33.20 (1.69)
Word Reading	20.40 (.87)	22.05 (1.19)	22.82 (1.36)	22.67 (.90)	23.23 (1.27)	25.75 (1.44)
Inhibition	43.85 (1.32)	51.47 (4.71)	49.47 (2.98)	58.44* (2.69)	66.05 (3.98)	70.20* (4.50)
Inhibition Switching	50.65 (1.71)	55.74 (2.30)	59.35 (5.21)	60.83 (4.00)	73.09 (4.66)	72.79 (6.85)
**Trails**						
Visual Scanning	20.20 (1.34)	17.26 (1.16)	18.82 (.79)	19.78 (1.16)	24.50 (1.66)	24.86 (1.56)
Number	27.00 (1.69)	23.63 (1.63)	27.82 (1.87)	29.56 (2.02)	41.14 (5.10)	41.38 (4.06)
Letter	25.45 (1.63)	26.16 (2.25)	28.00 (1.86)	32.56 (2.53)	43.32 (4.19)	40.05 (3.33)
Number-Letter Switching	60.95 (4.97)	55.84 (2.72)	65.82 (6.17)	84.00 (7.50)	114.32 (13.67)	105.86 (9.51)
Motor Speed	22.00 (1.71)	22.32 (.94)	26.18 (2.36)	25.22 (1.48)	32.77 (4.07)	35.14 (2.78)
**Design Fluency**						
Filled Dots	10.65 (.69)	11.68 (.90)	12.06 (1.02)	9.89* (.77)	10.09 (.96)	7.81 (.67)
Empty Dots	11.80 (9.55)	12.21 (.79)	13.59 (.78)	11.50**ss (1.04)	11.00 (.96)	9.24 (.76)
Switching	9.55 (.44)	9.58 (.67)	8.71 (.57)	8.39* (.63)	6.86 (.59)	7.10 (.61)
**Verbal Fluency**						
Letter	42.80 (2.79)	43.11 (2.60)	42.24 (3.07)	41.61 (3.18)	43.27 (2.67)	41.62 (2.97)
Category	44.95 (1.63)	46.21 (1.77)	43.47 (1.73)	43.72 (1.90)	39.22 (1.74)	41.76 (2.04)
Switching	15.55 (.56)	14.79 (.72)	15.82 (.61)	15.22 (.59)	13.27 (.63)	13.00 (.72)

*Note*. Significant exploratory analyses are denoted here using *** = p < .001;

** = p < .01;

* = p < .05.

**Table 9:** Relationship between age, metabolic status, learning and memory.

	Age Group & Metabolic Status: Mean (Standard Error)					
Variable	Young Control	Young Metabolic	Middle-age Control	Middle-age Metabolic	Older Control	Older Metabolic
**CVLT-II**						
Trial 1	6.70 (.38)	6.94 (.45)	7.12 (.47)	6.28 (.45)	6.05 (.59)	6.40 (.47)
Trial 2	10.05 (.54)	9.67 (.40)	10.24 (.53)	9.61 (.51)	8.45 (.60)	8.45 (.53)
Trial 3	12.05 (.51)	11.67 (.51)	11.53 (.52)	11.56 (.49)	9.77 (.69)	10.35 (.68)
Trial 4	12.90 (.48)	12.44 (.52)	12.82 (.52)	12.50 (.49)	10.86 (.65)	10.95 (.57)
Trial 5	12.85 (.45)	13.17 (.44)	13.00 (.58)	12.83 (.60)	11.05 (.59)	11.30 (.62)
Total Recall	54.55 (1.91)	53.89 (1.82)	54.71 (2.15)	52.78 (1.99)	46.18 (2.86)	47.45 (2.54)
Short Delay FR	11.85 (.59)	11.89 (.62)	12.41 (.69)	11.22* (.74)	9.32 (.80)	9.35 (.81)
Long Delay FR	12.15 (.73)	12.89 (.52)	13.12 (.61)	12.78 (.59)	10.50 (.72)	11.20 (.62)
Short Delay CR	12.55 (.69)	12.22 (.62)	12.76 (.76)	12.56 (.68)	9.50 (.73)	9.95 (.85)
Long Delay CR	12.65 (.65)	12.89 (.52)	13.18 (.69)	12.94 (.60)	10.23 (.77)	11.70 (.68)
**BVMT-R**						
Total Recall	27.50 (1.07)	27.22 (1.20)	24.13 (1.13)	22.76 (2.01)	20.57 (1.52)	20.35 (1.85)
Long Delay Recall	10.40 (.32)	10.22 (.41)	10.00 (.40)	8.82 (.75)	8.81 (.58)	8.20 (.75)

***Note***. CVLT-II = California Verbal Learning Test-II, FR = free recall, CR = cued recall, BVMT-R = Brief Visual Memory Test-Revised. Significant exploratory analyses are denoted here using *** = p < .001;

** = p < .01;

* = p < .05.

Exploratory analyses investigated the role of weight as a covariate when evaluating the possible influences of age group and metabolic status on cognitive functioning. Young adults demonstrated significant differences by metabolic status on Digit Span ([*F*(1,29) = 15.65, p < .01], Table 7) and CPT-2 Response time ([*F*(1,29) = 5.16, p = .03], Table 7) when controlling for weight in addition to gender and education level. In the middle-age group, these MANCOVAs revealed a significant relationship between metabolic status and performance on the D-KEFS Color-Word Inhibition ([*F*(1,34) = 6.00, p = .02], Table 8); CVLT-II Short Delay Free Recall ([*F*(1,34) = 5.49, p = .03], Table 9); D-KEFS Design Fluency: filled dots ([*F*(1,34) = 4.40, p = .04], Table 8), empty dots ([*F*(1,34) = 8.30, p < .01], Table 8), and switching ([*F*(1,34) = 5.10, p = .03], Table 8), when weight was added into the MANCOVA as a covariate. There were significant differences by metabolic status in the older adult group on the D-KEFS Color-Word Inhibition ([*F*(1,39) = 4.36, p = .04], Table 8) and CPT-2 Perseveratives ([*F*(1,33) = 5.03, p = .03], Table 7). There were not significant differences between metabolic status and cognitive performance when controlling for weight, gender, and education level for any other cognitive assessment in this study for young, middle-aged, or older adult age groups.

## Discussion

The primary aim of the current study was to investigate differences in cognitive functioning among young adults (classified as normal or at risk for MetS), middle-aged (classified as normal or MetS), and older adults (classified as normal or MetS).

### Self report measures

Individuals with MetS rated themselves as less inhibited and more hungry than controls, regardless of age, on the TFEQ, a self-report measure of eating behavior (Table 2). Additionally, middle-aged adults reported significantly less self-control than young and older adults on the BIS, a self-report measure of impulsivity (S1 Table). Obese persons have been found to report significantly more disinhibited eating than their normal weight counterparts [56, 57]. Disinhibition increases likelihood of weight gain and has been associated with a sedentary lifestyle [58, 59], which contribute to the development of MetS [60].

### Age group effects on cognitive performance

There were significant age group effects across neuropsychological assessments (Tables 3 and 4). Age-related cognitive decline has been consistently documented within the literature [61–68]. Of note, the present study showed age group effects across verbal and visual memory, executive functioning, and processing speed.

### Metabolic status effects on cognitive performance

There was a main effect of metabolic status on cognitive performance in which participants with MetS were significantly slower on the Color-Word Interference Test: Inhibition condition (Table 8, Fig 1). The Inhibition condition from the D-KEFS requires intact processing speed and cognitive flexibility [42]. It is interesting that, in the present cohort, individuals with MetS rated themselves as more disinhibited with eating as compared to normal participants; however, there were no significant correlations between self-reported disinhibition (*r* = .*137)* or self-control (*r* = .*034)* and performance on the Color-Word Interference: Inhibition task, when controlling for age (*r* = .136). Given the task demands of the Inhibition condition, this finding may provide support for declines in executive functioning abilities for individuals with MetS. Studies examining larger cohorts that incorporate executive functioning measures provide additional support for the present results [17, 18, 36].

Clinically, changes in executive functioning are associated with declines in activities of daily living and medication adherence [69–72]. In addition, comorbidity of vascular risk factors is also associated with functional decline [73] and declines in one’s ability to manage vascular risk factors [74]. Thus, an individual with MetS may be at risk for poor medication compliance which could exacerbate MetS. As a result, poor medication compliance could ultimately increase the risk of developing dementia.

### Metabolic status and age group effects on cognitive performance

Scores on measures of commission, response time, variability, perseveratives, and response style under the CPT-2 demonstrated significant interactions between age group and metabolic status. These interactions across the CPT-2 suggest that the young and middle-aged adults with MetS made more errors (commissions, perseveratives) and were more inconsistent in their responses as compared to controls. However, the older adults with MetS had fewer errors, better response times, and more consistency as compared to healthy older adults. Across age groups, participants with MetS also had faster response times (although this does not imply accuracy). These data support the notion of relative deficits related to attention in young and middle-aged adults with MetS. They also suggest the potential for a survivor effect or protective effect of MetS in older adults [35, 75, 76].

Due to the notable variability in weight in pounds for the middle-aged adults (Table 1), the extensive literature implicating obesity in cognitive dysfunction [25, 30, 77–84], and risk for dementia associated with weight in middle age [85–89], exploratory analyses were performed to assess the role of weight in cognition in the context of MetS. These analyses revealed significant differences on select executive functioning tasks (i.e., D-KEFS Color Word Interference: Inhibition and D-KEFS Design Fluency: Filled dots, Empty dots, and Switching subtests) between middle-aged adults with MetS versus control, with participant weight in pounds as a moderator. On each of these tasks, middle-aged control adults significantly outperformed those with MetS when weight in pounds was controlled for as an addition to gender and education level. These results are consistent with Yang and colleagues’ findings of deficits on executive functioning tasks for obese individuals and inhibition impairments for overweight individuals [75]. Of note, inherently poor executive functioning skills predicted obesity in longitudinal studies of children [30]. Based on these exploratory analyses, weight in pounds moderates the relationship between executive functioning on select tasks and MetS in middle-aged adults. This relationship likely highlights the compounding deleterious effects of excess weight and MetS on executive functioning in middle-aged adults in contrast with the purported protective effect of excess weight on cognition in older adults [35, 75]. However, this “protective effect” may potentially be partially accounted for by a survivor effect as MetS has been associated with increased mortality across the lifespan as compared to those without MetS [76].

Declines in executive functioning in MetS have been reported in the literature [17, 19, 31, 36, 90]. However, within these experiments executive functioning was assessed via screening measures or defined as a latent variable combining multiple processes such as novel problem solving, cognitive set-shifting, inhibition, and fluency. Based on the literature, it is difficult to determine which executive functioning processes are affected in MetS. In fact, primary criticisms in a review of the literature on cognitive performance in MetS were the “lack of consistency in the cognitive domains selected for assessment, differences in the quality of tests selected, and demographics of populations studied” [36]. Furthermore, a 2018 review examining the association between cognitive functioning and MetS concluded that the heterogeneity of results between studies was too great to infer MetS as a precursor to declines or changes in cognition [35]. As such, the present study adds to the literature through investigating the effects of MetS on individual tests of cognitive functioning.

There is a paucity of research regarding MetS in adolescents. One study found a significant negative impact of MetS on executive functioning and cognitive flexibility skills in Hispanic adolescents [91]. Additionally, adolescents with MetS demonstrated significantly poorer performances on measures of reading, attention, and working memory [92]. Obesity in adolescents has also been associated with executive dysfunction, decreased levels of inhibition and slower processing speed [26, 28, 93, 94]. When accounting for weight in this study, young adults at risk for MetS demonstrated significant differences on two tasks of attention (Digit Span and CPT-2 Response Time), in which those categorized as MetS performed significantly better than young controls. These results are inconsistent with the aforementioned literature and could be due to the small sample size. There were no effects of MetS on remaining measures of cognitive performance in the present study in this young adult sample (whether or not weight was accounted for).

## Limitations

Strengths and limitations of this work should be recognized. The strengths of this work lie in the thorough neuropsychological evaluation and the life-span approach to assessing the effects of metabolic syndrome on cognitive function. There are also limitations. First, the study would have benefitted from larger sample sizes. The young adult metabolic cohort was comprised of obese individuals who are at risk for the development of MetS. The study is cross sectional and the duration during which an individual met criteria for MetS was not available, which could also underestimate the effect of MetS. Future studies will be needed to determine the effect of duration of MetS on lifespan cognitive function. Finally, the sample was largely Caucasian, middle class, and, on average, had some college education, suggesting the need for future studies in more diverse cohorts.

## Conclusions

The current study investigated cognitive performance in a community sample of young, middle-aged and older adults with multiple risk factors for metabolic syndrome. Participants with MetS were significantly slower on the Color-Word Interference: Inhibition task as compared to controls. Middle-aged adults with MetS appeared to be more susceptible to executive functioning deficits with weight in pounds moderating this relationship. MetS in young and middle-aged adults may be associated with relative deficits in attention. Cognitive performance by older adults with MetS could suggest a survivor effect or protective effect of MetS. The results of the present study provide further evidence for age-related declines in cognitive functioning.

Innate executive dysfunction may be a causal factor in becoming obese [30]. While purely speculative, deficits in inhibition and executive function could potentially contribute to difficulties maintaining a healthy diet and adequate exercise, both of which contribute to the development and maintenance of MetS. Individuals with MetS self-report greater levels of disinhibited eating and hunger than controls, which may also have implications for the development and maintenance of MetS.

Given that individuals with MetS had significantly greater self-reported disinhibited eating and performed more poorly on a task of inhibition, future studies aimed at investigating potential causal relationships between MetS and disinhibited eating and executive dysfunction may provide insight into effective intervention targets to delay or prevent MetS.

## Supporting information

S1 Table Bonferroni pairwise comparisons. (DOCX)

S1 Data (XLSX)
