# Supplementary material for: Metabolic syndrome and cognitive performance across the adult lifespan
Source: PLoS One. 2021 May 6;16(5):e0249348. doi: 10.1371/journal.pone.0249348 (PMC8101918; doi:10.1371/journal.pone.0249348)
Supplement: S1 Table — (DOCX) [file pone.0249348.s001.docx]

**S1 Table.** Bonferroni Pairwise Comparisons

| Age Group Comparison | Contrast Estimate | Significance  *p* |
| --- | --- | --- |
| **BMI** | | |
| YA vs. MA | -3.92 | .00*** |
| YA vs. OA | -.07 | 1.00 |
| MA vs. OA | 3.85 | .00*** |
| **Weight in Pounds** | | |
| YA vs. MA | -22.10 | .01** |
| YA vs. OA | 11.70 | .26 |
| MA vs. OA | 33.80 | .00*** |
| **Barrat Impulsiveness Scale: Attention** | | |
| YA vs. MA | .40 | 1.00 |
| YA vs. OA | -.15 | 1.00 |
| MA vs. OA | -.55 | .93 |
| **Barrat Impulsiveness Scale: Motor** | | |
| YA vs. MA | -.85 | .60 |
| YA vs. OA | .43 | 1.00 |
| MA vs. OA | 1.28 | .16 |
| **Barrat Impulsiveness Scale: Self-Control** | | |
| YA vs. MA | -1.55 | .16 |
| YA vs. OA | .66 | 1.00 |
| MA vs. OA | 2.21 | .02* |
| **Barrat Impulsiveness Scale: Cognitive Complexity** | | |
| YA vs. MA | -.72 | .56 |
| YA vs. OA | -.58 | .84 |
| MA vs. OA | .15 | 1.00 |
| **Barrat Impulsiveness Scale: Perseverance** | | |
| YA vs. MA | -.83 | .15 |
| YA vs. OA | -.23 | 1.00 |
| MA vs. OA | .60 | .45 |
| **Barrat Impulsiveness Scale: Cognitive Instability** | | |
| YA vs. MA | .67 | .26 |
| YA vs. OA | .51 | .54 |
| MA vs. OA | -.16 | 1.00 |
| **TFEQ: Cognitive Restraint** | | |
| YA vs. MA | .40 | 1.00 |
| YA vs. OA | -1.34 | .61 |
| MA vs. OA | -1.74 | .30 |
| **TFEQ: Disinhibition** | | |
| YA vs. MA | -.65 | 1.00 |
| YA vs. OA | .47 | 1.00 |
| MA vs. OA | 1.13 | .45 |
| **TFEQ: Hunger** | | |
| YA vs. MA | -1.05 | .34 |
| YA vs. OA | -.02 | 1.00 |
| MA vs. OA | 1.02 | .36 |
| **MMSE** | | |
| YA vs. MA | .38 | .57 |
| YA vs. OA | .97 | .00** |
| MA vs. OA | .58 | .12 |
| **DRS Total Score** | | |
| YA vs. MA | .34 | 1.00 |
| YA vs. OA | .52 | 1.00 |
| MA vs. OA | .18 | 1.00 |
| **Digit Span Total** | | |
| YA vs. MA | 2.02 | .09 |
| YA vs. OA | 3.67 | .00*** |
| MA vs. OA | 1.64 | .21 |
| **BVMT-R Trials 1-3 Total** | | |
| YA vs. MA | 3.71 | .06 |
| YA vs. OA | 6.85 | .00*** |
| MA vs. OA | 3.14 | .14 |
| **BVMT-R Delay** | | |
| YA vs. MA | .83 | .49 |
| YA vs. OA | 1.78 | .01** |
| MA vs. OA | .94 | .32 |
| **CVLT-II Trials 1-5 Total** | | |
| YA vs. MA | -.12 | 1.000 |
| YA vs. OA | 6.40 | .01* |
| MA vs. OA | 6.49 | .01* |
| **CVLT-II Short Delay Free Recall** | | |
| YA vs. MA | -.14 | 1.00 |
| YA vs. OA | 2.23 | .00** |
| MA vs. OA | 2.37 | .00** |
| **CVLT-II Short Delay Cued Recall** | | |
| YA vs. MA | -.61 | 1.00 |
| YA vs. OA | 1.41 | .07 |
| MA vs. OA | 2.01 | .00** |
| **CVLT-II Long Delay Free Recall** | | |
| YA vs. MA | -.49 | 1.00 |
| YA vs. OA | 2.34 | .00** |
| MA vs. OA | 2.83 | .00*** |
| **CVLT-II Long Delay Cued Recall** | | |
| YA vs. MA | -.47 | 1.00 |
| YA vs. OA | 1.52 | 0.05 |
| MA vs. OA | 2.00 | 0.01* |
| **CPT-2 Omission** | | |
| YA vs. MA | 1.94 | 1.00 |
| YA vs. OA | -8.59 | .79 |
| MA vs. OA | -10.53 | 0.45 |
| **CPT-2 Commission** | | |
| YA vs. MA | 5.16 | .79 |
| YA vs. OA | -.23 | 1.00 |
| MA vs. OA | -5.40 | .70 |
| **CPT-2 Clinical Percentage** | | |
| YA vs. MA | -10.20 | .11 |
| YA vs. OA | -16.12 | .00** |
| MA vs. OA | -5.92 | .61 |
| **CPT-2 Non-Clinical Percentage** | | |
| YA vs. MA | 10.37 | .10 |
| YA vs. OA | 16.12 | .00** |
| MA vs. OA | 5.75 | .65 |
| **CPT-2 Variability** | | |
| YA vs. MA | 3.72 | 1.00 |
| YA vs. OA | -.59 | 1.00 |
| MA vs. OA | -4.31 | 1.00 |
| **CPT-2 Response Time** | | |
| YA vs. MA | -45.90 | .78 |
| YA vs. OA | -35.40 | 1.00 |
| MA vs. OA | -10.50 | 1.00 |
| **CPT-2 Perseverative** | | |
| YA vs. MA | -1.46 | 1.00 |
| YA vs. OA | -1.31 | 1.00 |
| MA vs. OA | .15 | 1.00 |
| **CPT-2 Response Style** | | |
| YA vs. MA | 2.83 | 1.00 |
| YA vs. OA | -3.90 | 1.00 |
| MA vs. OA | -6.73 | .73 |
| **D-KEFS Verbal Letter Fluency** | | |
| YA vs. MA | .16 | 1.00 |
| YA vs. OA | -.57 | 1.00 |
| MA vs. OA | -.73 | 1.00 |
| **D-KEFS Verbal Category Fluency** | | |
| YA vs. MA | 1.68 | 1.00 |
| YA vs. OA | 4.75 | .02* |
| MA vs. OA | 3.07 | .27 |
| **D-KEFS Verbal Fluency Switching Total** | | |
| YA vs. MA | -.54 | 1.00 |
| YA vs. OA | 1.86 | .01** |
| MA vs. OA | 2.40 | .00*** |
| **D-KEFS Verbal Fluency Switching Accuracy** | | |
| YA vs. MA | -.64 | 1.00 |
| YA vs. OA | 1.95 | .02* |
| MA vs. OA | 2.59 | .00** |
| **D-KEFS Design Fluency Filled Dots** | | |
| YA vs. MA | .19 | 1.00 |
| YA vs. OA | 2.10 | .04* |
| MA vs. OA | 1.91 | .08 |
| **D-KEFS Design Fluency Empty Dots** | | |
| YA vs. MA | -.57 | 1.00 |
| YA vs. OA | 2.03 | .05* |
| MA vs. OA | 2.59 | .01** |
| **D-KEFS Design Fluency Switching Total** | | |
| YA vs. MA | 1.00 | .33 |
| YA vs. OA | 2.59 | .00*** |
| MA vs. OA | 1.60 | .027 |
| **D-KEFS Design Fluency Switching Set Loss** | | |
| YA vs. MA | -.27 | 1.00 |
| YA vs. OA | -1.00 | .32 |
| MA vs. OA | -.73 | .73 |
| **D-KEFS Color-word Interference: Color Naming** | | |
| YA vs. MA | -2.22 | .44 |
| YA vs. OA | -3.98 | .024* |
| MA vs. OA | -1.75 | .74 |
| **D-KEFS Color-word Interference: Word Reading** | | |
| YA vs. MA | -1.33 | .83 |
| YA vs. OA | -3.28 | .02* |
| MA vs. OA | -1.94 | .33 |
| **D-KEFS Color-word Interference: Inhibition** | | |
| YA vs. MA | -5.80 | .36 |
| YA vs. OA | -20.60 | .00*** |
| MA vs. OA | -14.80 | .00*** |
| **D-KEFS Color-word Interference: Inhibition Switching** | | |
| YA vs. MA | -6.59 | .53 |
| YA vs. OA | -18.06 | .00*** |
| MA vs. OA | -11.47 | 0.05 |
| **D-KEFS Color-word Interference: Inhibition Errors** | | |
| YA vs. MA | .23 | 1.00 |
| YA vs. OA | -.53 | .73 |
| MA vs. OA | -.76 | .31 |
| **D-KEFS Color-word Interference: Inhibition Switching Errors** | | |
| YA vs. MA | -.11 | 1.00 |
| YA vs. OA | -1.24 | .03* |
| MA vs. OA | -1.2105 | .06* |
| **D-KEFS Trails: Visual Scanning** | | |
| YA vs. MA | -.43 | 1.00 |
| YA vs. OA | -5.82 | .00*** |
| MA vs. OA | -5.39 | .00*** |
| **D-KEFS Trails: Number Sequencing** | | |
| YA vs. MA | -3.31 | 1.00 |
| YA vs. OA | -16.12 | .00*** |
| MA vs. OA | -12.81 | .00*** |
| **D-KEFS Trails: Letter Sequencing** | | |
| YA vs. MA | -4.45 | .44 |
| YA vs. OA | -16.10 | .00*** |
| MA vs. OA | -11.65 | .00*** |
| **D-KEFS Trails: Number-Letter Switching** | | |
| YA vs. MA | -15.50 | .26 |
| YA vs. OA | -51.90 | .00*** |
| MA vs. OA | -36.40 | .00*** |
| **D-KEFS Trails: Motor Speed** | | |
| YA vs. MA | -3.62 | .54 |
| YA vs. OA | -12.20 | .00*** |
| MA vs. OA | -8.58 | .00** |

*Note.* YA = young adult, MA = middle-aged adult, OA = older adult.***** = p<.001; ** = p<.01; * = p<.05.
